# European countries policy responses against SARS-Cov-2 in the context of vaccinations

**DOI:** 10.1038/s41598-025-11881-3

**Published:** 2025-08-18

**Authors:** Arianna Agosto, Paola Cerchiello, Siegfried Eisenberg, Thomas Czypionka

**Affiliations:** 1 Dep. of Economics and Management, University of Pavia, 27100 Pavia, Italy; 2 Institute for Advanced Studies, Research Group Health Systems and Health Policy, Vienna, Austria; 3 Department of Health Policy, London School of Economics, London, UK

**Keywords:** COVID-19 pandemics, Country measures, Non-pharmaceutical interventions, Vaccination, Principal component analysis, Health care, Computational science, Scientific data, Statistics

## Abstract

Non-pharmaceutical interventions (NPIs) are essential tools for containing or mitigating the spread of a novel virus until vaccination becomes available. Given their well-known side effects, NPIs should be employed only as long as necessary and largely replaced by population immunity through vaccination. During the SARS-CoV-2 pandemic, countries adopted various strategies for implementing NPIs and administering vaccinations. While differences in NPIs and vaccination strategies among countries have been descriptively illustrated, they have not yet been quantified. This study aims to quantitatively analyze the differences in NPIs across 10 European countries immediately after vaccinations became available.

## Introduction

Non-pharmaceutical interventions (NPIs) are the initial tools for governments to mitigate the occurrence of new communicable diseases and can be considered as the main public health measures at an early stage of a pandemic to prevent populations from the burden of disease until pharmaceutical interventions are available, e.g., drugs or vaccinations^1^. There is a broad range of NPIs that differ in terms of their effectiveness but also in how much they infringe upon individuals’ freedom as well as their economic cost^2,3^.

Empirical studies investigating the effectiveness of NPIs in reducing the burden of SARS-Cov-2 were published right after data about NPIs and epidemiological parameters, e.g., incidence, reproduction number, were made available to offer policy recommendations about the most appropriate measures. Indeed, one year later the first systematic reviews were published^4–6^. Papers about vaccine effectiveness (VE) in terms of reduced infection rates and lower probabilities of severe disease were published shortly after the first doses were administered. Consequently, studies investigating the impact of different vaccines and number of vaccine doses^7^ among different population groups^8,9^, waning immunity and effectiveness against different virus variants^10,11^ were published in the following months. In addition to the first availability of vaccines and different vaccination strategies (e.g. duration between first and second dose), countries applied NPIs to different degrees and in different combinations^12–14^, while the understanding of the virus with respect to variants, vaccine effectiveness and measures increased over time^15^. Some studies^16^ also investigated the patterns in the evolution of the pandemic focusing solely on the dynamics of the reproduction rate (R_t) or the preparedness at the country level in terms of reaction to health related threats^17^.

Combinations of NPIs appeared to be more successful than individual measures, and it was highlighted that acceptance and compliance, which are key for measures to be effective^6^, differ within population groups. Additionally, it was revealed that the efficacy of NPIs differed among countries, which can be further explained by country characteristics^18^ as well as different timings of implementation^13^. The employment and consequently the effectiveness of NPIs decreased over time from the first to the third wave^19^. In the early months of the SARS-CoV-2 pandemic in 2020, most European countries responded with strict measures, such as lockdowns and the cancellation of public events^14^. However, in the second half of 2020, many countries relaxed their NPIs before the subsequent rise in SARS-CoV-2 cases during the winter of 2020/2021^20^. From that point onward, with the introduction of vaccination, countries diversified their strategies: some reintroduced NPIs, others employed only a few or limited them, while some abolished them entirely. In any case, the NPIs capacity of limiting the spread of the virus remained unaltered but rather the effects changed due to their limited implementation as to reduce the well-known social impacts.

In the European context of generally open border policies, the measures put in place by one country might affect the effectiveness of measures in geographically close countries. However, apart from different tools that descriptively illustrate government responses countrywide, e.g., Oxford Government Response Tracker^21^ or CoronaNet^22^, there is no proper statistical comparison of NPIs including administered vaccinations between European countries that reveals which country rely on which measure in contrast to other countries and at which period of the pandemic. One reason might be the complexity of comparing several measures and administered vaccinations across multiple countries. The present study aims to shed some light on this aspect, by analyzing and comparing the different countries’ strategies in terms of both NPIs and vaccination and their change in time.

Furthermore, the scientific community has been trying to disentangle the possible nexus between the several adopted NPIs and the role of vaccination. In doing so, researchers have faced the issue of endogeneity that is inherently ingrained in the considered variables. Indeed, the single policy measures and vaccination roll-out are strictly interconnected and correlated as the common latent trait is represented by the pandemic evolution which inevitably drives the increase or decrease of the countermeasures envisaged by countries. When endogeneity occurs, it is not possible to directly employ standard techniques and estimate predictive models without appropriately addressing it.

The present study focuses on the variation in country response strategies concerning NPIs and vaccination roll-out after the first vaccines against SARS-Cov-2 were available in 2021.

The objectives are:

- to generally investigate which measures and relative combinations were responsible the most for differences in national strategies over time;
- to reveal which European countries relied on stricter measures and what are these measures in contrast to other European countries;
- to investigate the role of vaccination with regard to NPIs.

Thus, we rely on a proper statistical model to exploit the latent variables structure underlying the countries’ reaction patterns. In particular, we employ Principal Component Analysis (PCA) to analyze the different country strategies and to disentangle the application of NPIs and vaccination roll-out, providing an effective representation of the diversified country responses in Europe. Evidence is expected to support the claim for more homogeneous health strategies in Europe by revealing that the heterogeneous approaches during the pandemic^23^, in the end, most likely are one of the main reasons for different excess mortality rates associated with SARS-Cov-2 across European countries^24,25^.

## Results

### Preliminary correlation analysis

Table 1 reports the values of correlation between the variables used in our empirical study. The correlation values are calculated on the whole dataset, so they can be considered as average values over the entire analyzed period. It is important to assess the correlation structure, since PCA depends heavily upon it. It can be noticed from Table 1 that nearly all correlations are statistically significant. The fact that all NPIs are positively correlated with each other can be interpreted as they are used complementary, rather than supplementary, in the country strategies. The highest correlation values are found between Social Distancing (SOC) and Business Restrictions (BUS) (0.726) and between the latter and Mask Usage (MSK) (0.606). It should also be noticed that the vaccination variable (number of administered vaccine doses per million people, VAX) is significantly and positively correlated with some of the considered NPIs (SOC, BUS and MSK), but with a relatively small magnitude. This is likely connected to the different timing of vaccination strategies and restrictive measures. It is also interesting to point out the low average value of correlations induced by the Health Monitoring variable (HTM). Indeed, HTM cannot be considered a proper restriction strategy, but rather a monitoring tool used by all governments to assess the virus spread in the population regardless of the containment strategies put in place.

Nonetheless, it is relevant to stress that the observed significant correlations among the considered measures call for an appropriate statistical approach to exploit such an interdependence structure without incurring in endogeneity problems. As already mentioned in the introduction, PCA can be particularly useful in leveraging upon linear correlations patterns much better than classical supervised statical models like linear regression, logistic regression or opaque tree-based models. Indeed, this happens because PCA calls for highly correlated input variables as to create new features able to exploit the latent underlying structure. More details about PCA are provided in section Methods.

**Table 1:** Correlation matrix of the variables used in the study. The asterisk ($^*$) denotes statistical significance of the correlation coefficient of at least the 5% level. The highest and the lowest correlations are indicated in bold and italic color respectively.

	VAX	SOC	SCH	BUS	MSK	HTM
VAX	1.000*	0.265*	0.021	0.261*	0.170*	0.005
SOC	0.265*	1.000*	0.561*	0.726*	0.483*	-0.049*
SCH	0.021	0.561*	1.000*	0.536*	0.386*	0.125*
BUS	0.261*	0.726*	0.536*	1.000*	0.606*	0.061*
MSK	0.170*	0.483*	0.386*	0.606*	1.000*	0.168*
HTM	0.005	-0.049*	0.125*	0.061*	0.168*	1.000*

### Results of PCA analysis

**Fig. 1:**
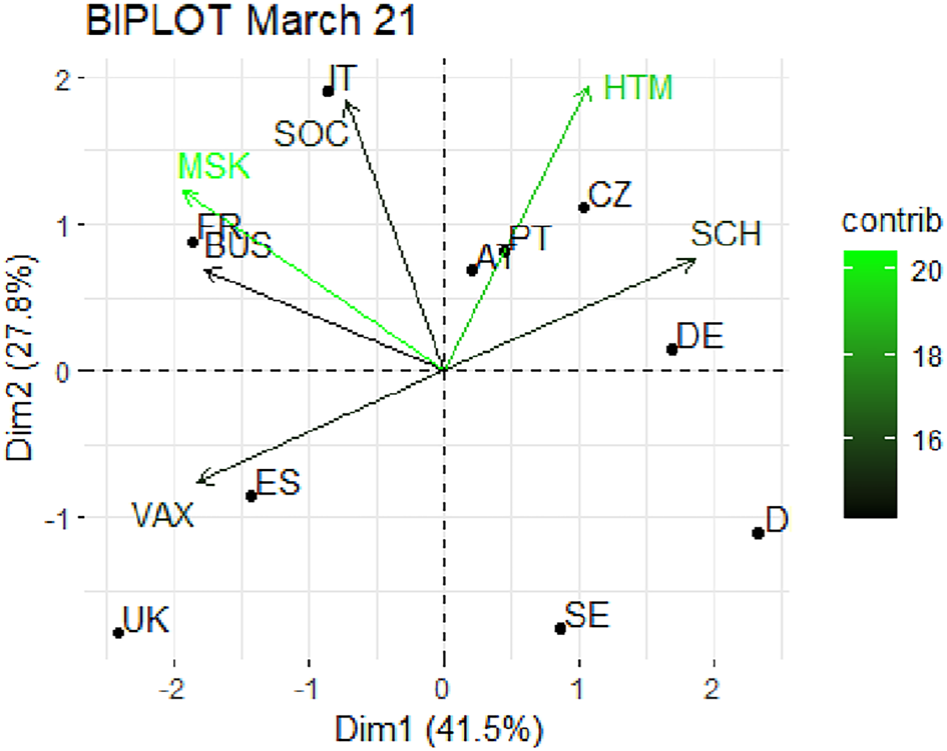
Biplot for March 2021: the data points are the countries while the arrows refer to the variables. The length and colour shades of the arrows are proportional to the contributions (in percentage) of the variables to the principal components. The contribution of a variable to a given principal component is (in percentage): (cos2 * 100) / (total cos2 of the component) where cos2 is calculated as the squared coordinates.

From Fig. 1 through Fig. 10, we report the biplots for each month associated with the corresponding fitted PCA. In our biplot representation, the data points are the countries, while the arrows refer to the policy and vaccination measures that contribute to the two components. Each biplot refers to a different month of the year 2021, from March to December. To further understand the role played by each considered variable regarding the two principal components, from Table 2 through Table 6 we report the loading values associated with the ten performed PCA. The loadings are the weights assigned to each variable with regards to each component and measure the contribution (importance) of that variable in explaining the specific component: the larger the weight, in the range [-1 ; +1], the stronger the impact. Additionally, Table 7 and Table 8 summarise the loadings for each measure over time, and Table 9 reveals which measures countries mostly relied on.

**Fig. 2:**
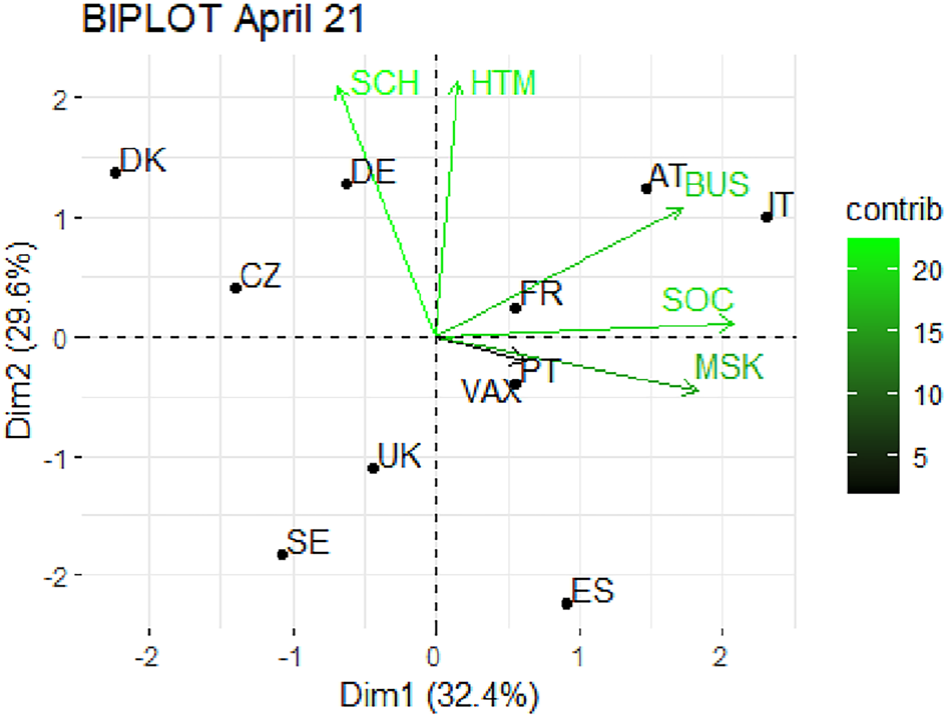
Biplot for April 2021: the data points are the countries while the arrows refer to the variables. The length and colour shades of the arrows are proportional to the contributions (in percentage) of the variables to the principal components. The contribution of a variable to a given principal component is (in percentage) : (cos2 * 100) / (total cos2 of the component) where cos2 is calculated as the squared coordinates.

In Figs. 1 and 2, we provide the PCA representation for March and April 2021 respectively. As for March, we find that the first two components cumulate almost 70% of the total variability, signaling a good model performance. It can be noticed from Table 2 that the first component is mainly driven by VAX, BUS, MSK and SCH, with absolute values between 0.45 and 0.49; while the second is mainly connected to SOC and HTM with loadings of 0.57 and 0.61, respectively. The distribution of the countries in the biplot is signaling a quite diversified approach to the emergency after the first pandemic year. In particular, Italy appears more expressed towards SOC, while United Kingdom is more focused on VAX. Czechia, Portugal and Austria are more active in HTM and SCH, France instead in MSK and BUS. On the contrary, in the bottom right quadrant, Sweden and Denmark stand out for not adopting particularly restrictive measures.

In April 2021 (Fig. 2), we first notice a lower level of total explained variability, around 62%, for the two main components, and still a scattered pattern in the distribution of countries. In particular, Sweden and United Kingdom turn out to be the least expressed countries in terms of countermeasures adoption. Looking at the variable contribution, the first component is mainly associated with SOC, BUS and MSK, while the second goes with SCH and HTM (see Table 2). We also stress that, in this month, apart from Portugal, the vaccination roll-out seems not to contribute much to the variability of national strategies. Indeed, the vector associated with VAX has a short length, and the loadings expressing the variable contribution are low for both components.

**Fig. 3:**
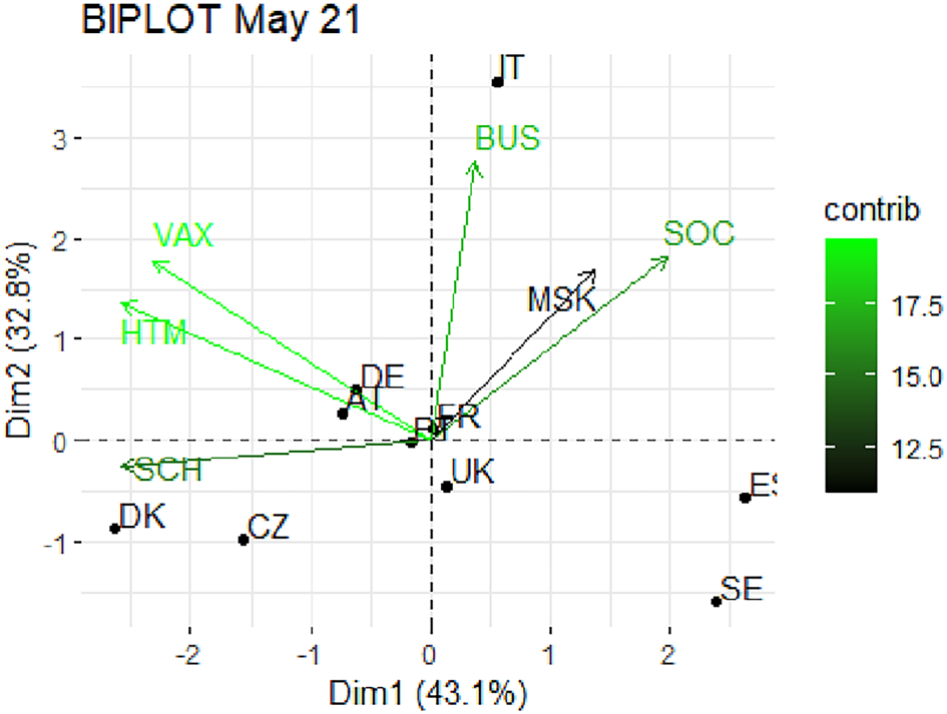
Biplot for May 2021: the data points are the countries while the arrows refer to the variables. The length and colour shades of the arrows are proportional to the contributions (in percentage) of the variables to the principal components. The contribution of a variable to a given principal component is (in percentage) : (cos2 * 100) / (total cos2 of the component) where cos2 is calculated as the squared coordinates.

**Fig. 4:**
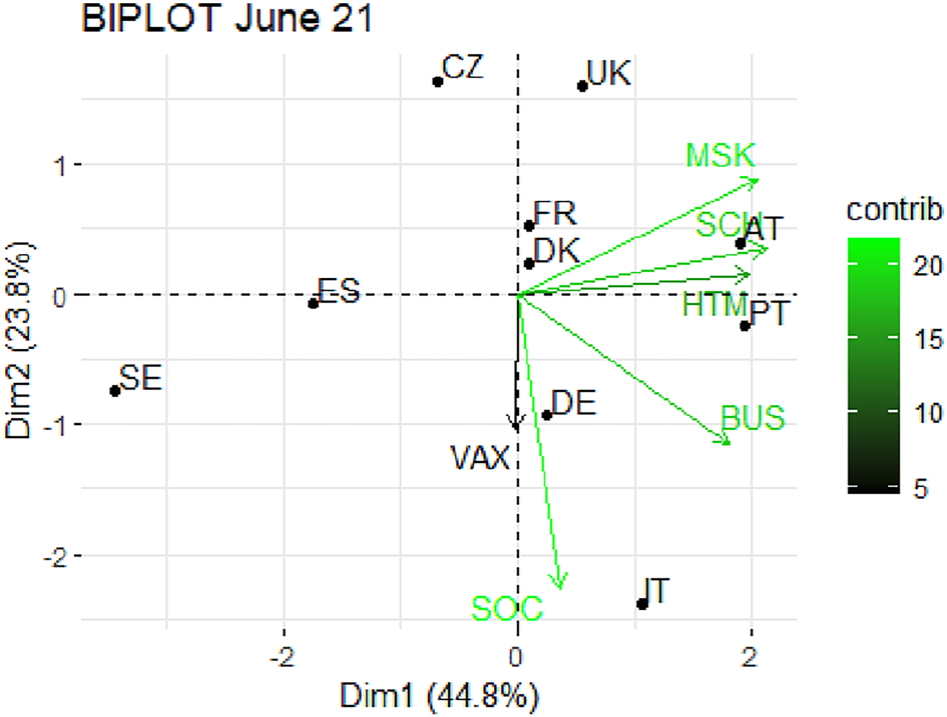
Biplot for June 2021: the data points are the countries while the arrows refer to the variables. The length and colour shades of the arrows are proportional to the contributions (in percentage) of the variables to the principal components. The contribution of a variable to a given principal component is (in percentage) : (cos2 * 100) / (total cos2 of the component) where cos2 is calculated as the squared coordinates.

Figures 3 and 4 show the output of PCA for May and June 2021 respectively. In May 2021, the total explained variance is almost 76%, decisively increased to the previous month, and the first component is mainly driven by VAX, SCH and HTM (see Table 3). The second component is instead connected with BUS, SOC and, again, VAX. The fact that, in this period, the vaccination variable is present in both components is likely due to the low variability of the variable itself. In other words, in May 2021 we do not see a distinctive role of vaccination versus the other countermeasures, different from what we will see in the following months. Further, we notice a cluster of 5 countries - United Kingdom, Portugal, France, Germany and Austria - laying on a central position. This means that those countries are showing an average profile for all the considered variables, without any specific trait. On the contrary, Italy is particularly severe in applying BUS and Denmark in SCH.

In June 2021 (Fig. 4), the percentage of explained variability is equal to 69%, with the first component mainly related to SCH, MSK, HTM and BUS, while the second component is driven by SOC. The country distribution is still scattered around and vaccination seems not to play a crucial role yet. Italy stands out again for adopting a particularly severe SOC and Austria for SCH and MSK, while Sweden, Czechia and United Kingdom place themselves at the margin of the biplot, signaling a general low restrictions behavior in managing the pandemic.

**Fig. 5:**
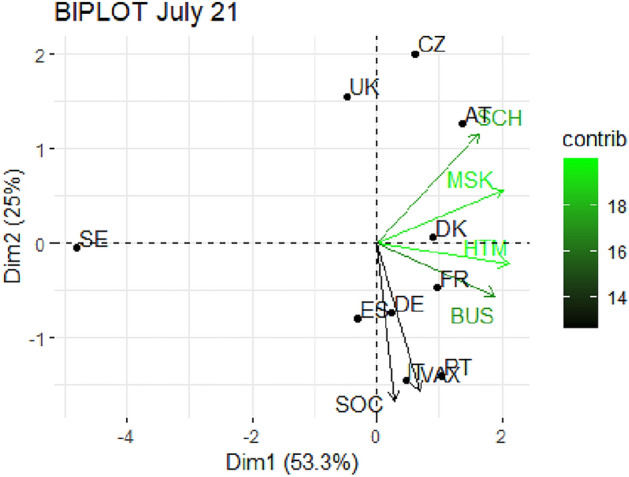
Biplot for July 2021: the data points are the countries while the arrows refer to the variables. The length and colour shades of the arrows are proportional to the contributions (in percentage) of the variables to the principal components. The contribution of a variable to a given principal component is (in percentage) : (cos2 * 100) / (total cos2 of the component) where cos2 is calculated as the squared coordinates.

**Fig. 6:**
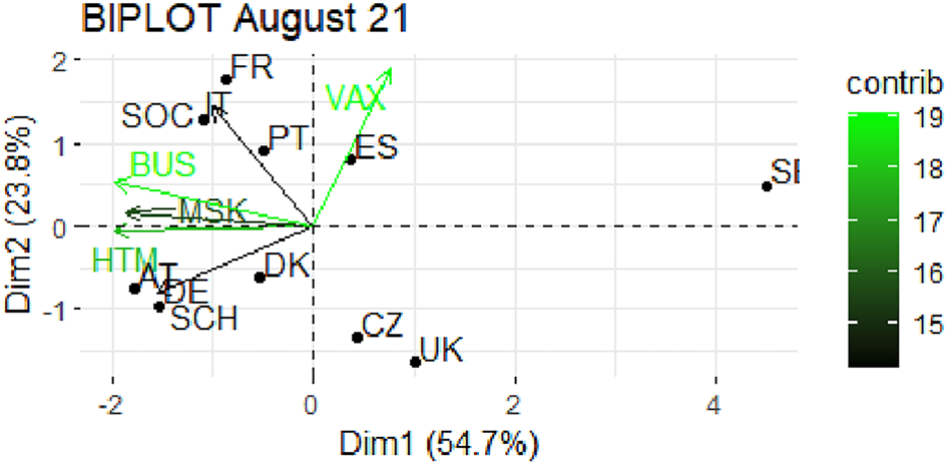
Biplot for August 2021: the data points are the countries while the arrows refer to the variables. The length and colour shades of the arrows are proportional to the contributions (in percentage) of the variables to the principal components. The contribution of a variable to a given principal component is (in percentage) : (cos2 * 100) / (total cos2 of the component) where cos2 is calculated as the squared coordinates.

In Figs. 5 and 6, we report the biplot representation for July and August 2021 respectively. In July, the two first components cumulate more than 78% of explained variance, a particularly high quota. Moreover, we notice the beginning of an interesting pattern that will consolidate in the following months. Indeed, the second component starts being strongly correlated with VAX (see Table 4), while the first component is mainly led by restrictive measures. Italy, Portugal, Spain and Germany are particularly expressed with the second component, while, once again, Sweden, United Kingdom and Czechia are at the margin. The same pattern consolidates in August 2021 (Fig. 6), where the two components cumulate 78.5 % of variability and the second component is even more connected with the vaccination. The distribution of the countries is interesting, as we clearly spot a cluster of more stringent countries composed by France, Italy, Portugal and Spain, as opposed to those more reluctant in imposing restrictions like United Kingdom, Czechia, Sweden and, in part, Germany, Denmark and Austria.

**Fig. 7:**
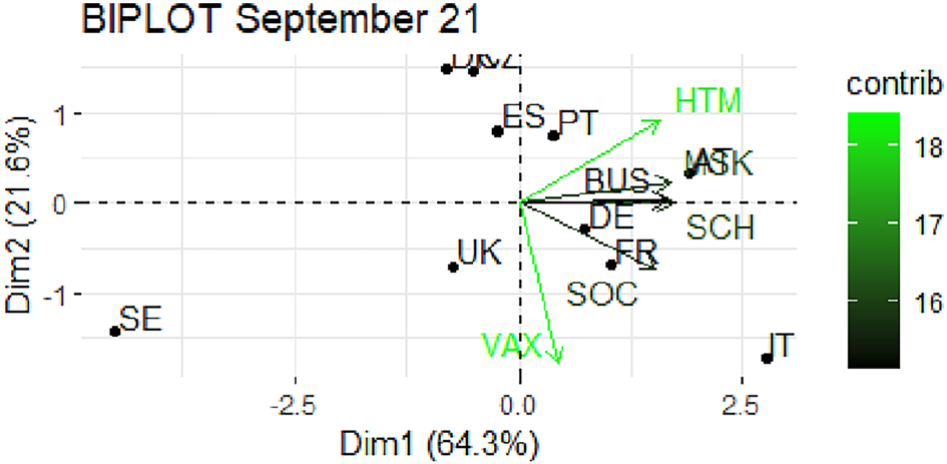
Biplot for September 2021: the data points are the countries while the arrows refer to the variables. The length and colour shades of the arrows are proportional to the contributions (in percentage) of the variables to the principal components. The contribution of a variable to a given principal component is (in percentage) : (cos2 * 100) / (total cos2 of the component) where cos2 is calculated as the squared coordinates.

**Fig. 8:**
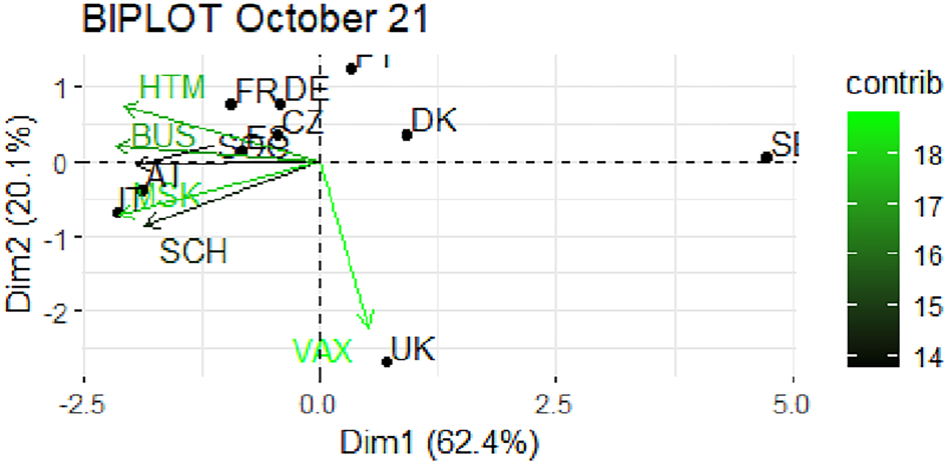
Biplot for October 2021: the data points are the countries while the arrows refer to the variables. The length and colour shades of the arrows are proportional to the contributions (in percentage) of the variables to the principal components. The contribution of a variable to a given principal component is (in percentage) : (cos2 * 100) / (total cos2 of the component) where cos2 is calculated as the squared coordinates.

In Figs. 7 and 8, we report the biplots referred to September and October 2021 respectively. The cumulative explained variance is even higher now, reaching values above 82%, and the second component is nearly totally driven by the vaccination variable (see Table 5). The first component instead represents a combination of all the stringency measures with similar weights. Denmark, Czechia and Spain seem not to focus on vaccination and show restrictions fairly below the average. On the opposite, the remaining countries proceed with their strategy of using different NPIs. During October 2021, the two components reach more than 82% of explained variability and vaccination is even more related to the second component (with a loading equal to 0.85, the highest ever). The polarization between stringency measures and vaccination has reached a peak. Most countries lay on the top quadrant of the biplot, which, in this case, is associated with low levels of vaccination and lower restrictions. In contrast to this, Austria and Italy rely on MSK and SCH. Once again, United Kingdom and Sweden have extreme patterns: the former seems to push again on vaccination only, while the latter is not expressed in vaccination and is extremely low in restrictions.

**Fig. 9:**
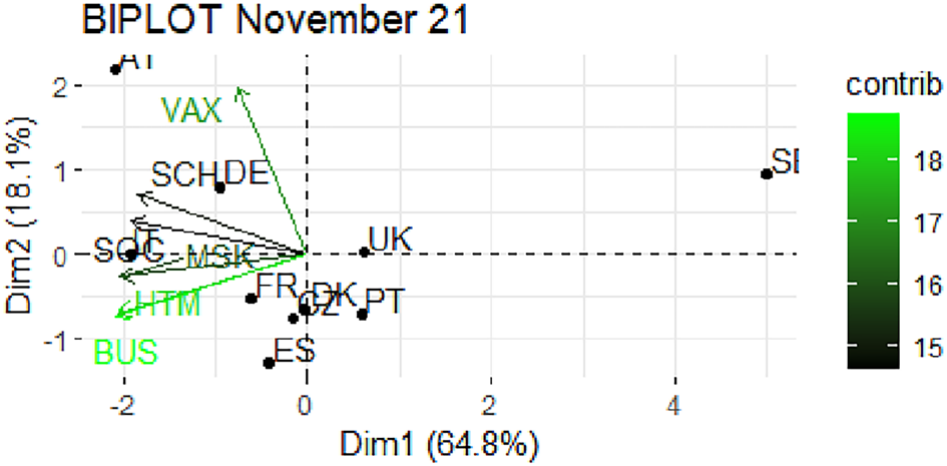
Biplot for November 2021: the data points are the countries while the arrows refer to the variables. The length and colour shades of the arrows are proportional to the contributions (in percentage) of the variables to the principal components. The contribution of a variable to a given principal component is (in percentage) : (cos2 * 100) / (total cos2 of the component) where cos2 is calculated as the squared coordinates.

**Fig. 10:**
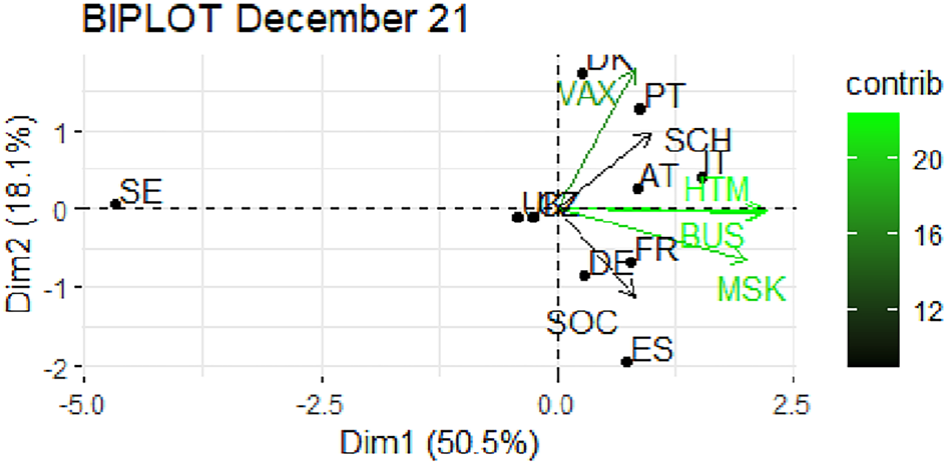
Biplot for December 2021: the data points are the countries while the arrows refer to the variables. The length and colour shades of the arrows are proportional to the contributions (in percentage) of the variables to the principal components. The contribution of a variable to a given principal component is (in percentage) : (cos2 * 100) / (total cos2 of the component) where cos2 is calculated as the squared coordinates.

Finally, in Figs. 9 and 10, we report the PCA results for the last two analyzed months, November and December 2021 respectively. The former shows a total explained variability above 80%, with the two components having the same characteristics as in the previous two months (see Table 6). There is again a cluster of countries fairly expressed on restrictions and very low on vaccinations. The most noticeable countries are Austria and Germany, strongly focused on vaccination, and Italy relying on SOC. The remaining countries go ahead without any strong restrictions. Finally, in December, we observe a lower cumulative total variability, below 70%, and a similar polarization of the two components, the first related to restrictions, in particular BUS, MSK and HTM, and the second to VAX. In this last scenario, the countries in extreme positions are Sweden, with weak restrictions and average vaccinations, and Austria and Italy with strict SCH and BUS restrictions and HTM. Denmark and Portugal are strong in VAX while Spain, Czechia and United Kingdom are poorly expressed on VAX.

**Table 2:** Variable loadings from the PCA analysis: March and April 2021.

Mar. 2021	PC1	PC2	Apr. 2021	PC1	PC2
VAX	$-0.466$	$-0.240$	VAX	0.185	$-0.062$
SOC	$-0.184$	0.573	SOC	0.613	0.034
SCH	0.474	0.236	SCH	$-0.203$	0.651
BUS	$-0.453$	0.213	BUS	0.509	0.333
MSK	$-0.494$	0.380	MSK	0.537	$-0.139$
HTM	0.274	0.607	HTM	0.043	0.664

**Table 3:** Variable loadings from the PCA analysis: May and June 2021.

May 2021	PC1	PC2	Jun. 2021	PC1	PC2
VAX	-0.467	0.412	VAX	-0.007	-0.356
SOC	0.400	0.419	SOC	0.089	-0.780
SCH	-0.520	-0.059	SCH	0.530	0.120
BUS	0.076	0.634	BUS	0.452	-0.394
MSK	0.278	0.389	MSK	0.511	0.304
HTM	-0.519	0.313	HTM	0.496	0.052

**Table 4:** Variable loadings from the PCA analysis: July and August 2021.

Jul. 2021	PC1	PC2	Aug. 2021	PC1	PC2
VAX	0.173	$-0.580$	VAX	0.195	0.736
SOC	0.074	$-0.620$	SOC	$-0.259$	0.561
SCH	0.417	0.432	SCH	$-0.401$	$-0.312$
BUS	0.484	$-0.206$	BUS	$-0.503$	0.204
MSK	0.516	0.211	MSK	$-0.475$	0.067
HTM	0.539	$-0.081$	HTM	$-0.506$	$-0.022$

**Table 5:** Variable loadings from the PCA analysis: September and October 2021.

Sep. 2021	PC1	PC2	Oct. 2021	PC1	PC2
VAX	0.121	$-0.829$	VAX	0.116	$-0.854$
SOC	0.417	$-0.340$	SOC	$-0.426$	$-0.015$
SCH	0.465	0.002	SCH	$-0.403$	$-0.327$
BUS	0.449	0.016	BUS	$-0.473$	0.080
MSK	0.461	0.111	MSK	$-0.463$	$-0.274$
HTM	0.426	0.430	HTM	$-0.453$	0.284

**Table 6:** Variable loadings from the PCA analysis: November and December 2021.

Nov. 2021	PC1	PC2	Dec. 2021	PC1	PC2
VAX	$-0.167$	0.830	VAX	0.205	0.737
SOC	$-0.424$	0.160	SOC	0.207	$-0.468$
SCH	$-0.410$	0.298	SCH	0.247	0.405
BUS	$-0.462$	$-0.307$	BUS	0.543	$-0.020$
MSK	$-0.451$	$-0.114$	MSK	0.505	$-0.272$
HTM	$-0.456$	$-0.298$	HTM	0.552	$-0.010$

In general, from a country perspective, as shown in Table 9, Austria and Italy represent the countries that relied the most on NPIs in 2021. Germany, France and Portugal relaxed their mix of measures in spring and autumn and focused on VAX and SOC in December. The remaining countries implemented, at most, conspicuous measures in specific periods. Among these, Denmark, Spain, and Czechia stood out at single months with respect to NPIs, while United Kingdom strategy was mainly focused on vaccinations. A peculiar case is Sweden, which never relied heavily on policy measures.

**Table 7:** Variable weights (loadings) for PC1. The highest positive and negative values are highlighted in bold and italic respectively.

Measure	PC1									
	March	April	May	June	July	August	September	October	November	December
VAX	*-0.466*	0.185	*-0.467*	-0.007	0.173	0.195	0.121	0.116	-0.167	0.205
SOC	-0.184	**0.613**	**0.400**	0.089	0.074	-0.259	**0.417**	*-0.426*	*-0.424*	0.207
SCH	**0.474**	-0.203	*-0.520*	**0.530**	**0.417**	*-0.401*	**0.465**	*-0.403*	*-0.410*	0.247
BUS	*-0.453*	**0.509**	0.076	**0.452**	**0.484**	*-0.503*	**0.449**	*-0.473*	*-0.462*	**0.543**
MSK	*-0.494*	**0.537**	0.278	**0.511**	**0.516**	*-0.475*	**0.461**	*-0.463*	*-0.451*	**0.505**
HTM	0.274	0.043	*-0.519*	**0.496**	**0.539**	*-0.506*	**0.426**	*-0.453*	*-0.456*	**0.552**

**Table 8:** Variable weights (loadings) for PC2. The highest positive and negative values are highlighted in bold and italic respectively.

Measure	PC2									
	March	April	May	June	July	August	September	October	November	December
VAX	-0.240	-0.062	**0.412**	-0.356	*-0.580*	**0.736**	*-0.829*	*-0.854*	**0.830**	**0.737**
SOC	**0.573**	0.034	**0.419**	*-0.780*	*-0.620*	**0.561**	-0.340	-0.015	0.160	*-0.468*
SCH	0.236	**0.651**	-0.059	0.120	**0.432**	-0.312	0.002	-0.327	0.298	**0.405**
BUS	0.213	0.333	**0.634**	-0.394	-0.206	0.204	0.016	0.080	-0.307	-0.020
MSK	0.380	-0.139	0.389	0.304	0.211	0.067	0.111	-0.274	-0.114	-0.272
HTM	**0.607**	**0.664**	0.313	0.052	-0.081	-0.022	**0.430**	0.284	-0.298	-0.010

**Table 9:** Overview of the main relevant measures by country according to PCA analysis.

Month	Countries									
	Austria	Italy	Germany	France	Portugal	Denmark	Spain	Czechia	UK	Sweden
March	HTM, SCH	SOC	SCH	MSK, BUS	HTM, SCH	–	VAX	HTM, SCH	VAX	–
April	BUS	BUS	SCH	BUS, SOC	VAX, MSK	–	–	–	–	–
May	–	BUS	–	–	–	SCH	–	–	–	–
June	MSK, SCH	SOC	–	–	–	–	–	–	–	–
July	MSK, SCH	SOC	SOC	BUS	VAX	MSK, HTM	SOC	–	–	–
August	SCH	SOC	SCH	SOC	SOC	SCH	VAX	–	–	–
September	MSK, HTM	SOC, VAX	SOC	SOC	HTM	–	–	–	–	–
October	MSK, SCH	MSK, SCH	–	–	–	–	HTM, BUS	HTM	VAX	–
November	VAX	SOC	VAX	–	–	–	–	–	–	–
December	SCH, BUS, HTM	SCH, BUS, HTM	SOC	SOC	VAX	VAX	–	–	–	–

## Methods

### Data

We used time series data from the OurWorldInData data repository (https://github.com/owid/covid-19-data/tree/master/public/data ) for 10 European countries: Austria, Czechia, Germany, Denmark, Spain, France, Italy, Portugal, Sweden and United Kingdom. Countries were chosen to cover a large part of the European population and, as data quality allowed, all areas of the European continent. Our data cover the period ranging from 1 March 2021 to 31 December 2021, to account for the impact of national vaccination roll-out, which started between the end of 2020 and the beginning of 2021 in most countries.

From the same dataset, we also retrieved, for each country, the daily time series of the number of administered vaccine doses per million people, already smoothed by a 7-day-average to account for the day-of-week effect. By using the number of administered doses, we take into account the speed of vaccination roll-out, independent of the dose number (first or following doses) and the timing between different doses. Further, booster (third) doses, which had been specifically relevant in autumn and winter in 2021, are included by using this variable.

We then consider the following variables referred to restrictive measures:

- Social Distancing (SOC);
- School Restrictions (SCH);
- Business Restrictions (BUS);
- Health Monitoring (HTM);
- Mask Usage (MSK).

To obtain continuous values between 0 and 1 for the NPIs out of the ordinal Oxford Government Response Tracker data, we build the above-mentioned NPI variables by applying the methodology described in^26^. Specifically, Table 10 shows how the considered NPIs and vaccination metrics were calculated using the variables available in the OurWorldInData repository.

**Table 10:** Calculation of the NPI variables used in the study.

Countermeasure	Input variables
Social distancing (SOC)	mean(C3_Cancel.public.events, C4_Restrictions.on.gatherings, C51_Close.public.transport,
	C6_Stay.at.home.requirements, C7_Restrictions.on.internal.movement)
School restrictions (SCH)	C1_School.closing
Business restrictions (BUS)	C2_Workplace.closing
Health monitoring (HTM)	H2_Testing.policy
Masks (MSK)	H6_Facial.Coverings
New vaccinations (VAX)	new_vaccinations_smoothed_per_million

### Analysis

Principal component analysis (*PCA*) aims at creating new components from a larger set of observed variables *Y*, where each component is a linear combination of the *Y* original variables as described in^27^. The model can be represented by the following equation:

1 $$\begin{aligned} C_1=w_{1}Y_{1}+\ldots +w_{K}Y_{K} \end{aligned}$$

where $C_1$ is the new first principal component obtained as the linear combination of $Y_i$ that are the original variables and $w_i$ that are the weights of the combination. The remaining $C_k$ components are built similarly, with *k* ranging between 1 and *K*, where *K* is the total number of variables.

According to the definition, PCA aims at finding new and linear-wise combinations of the original data, in a way that the amount of explained variance of the data is maximized. Those combinations are mathematically constrained to be mutually orthogonal (that is independent) and are called Principal Components (PC) or loadings. Given a $n\times k$ data matrix ${\bf {X}}$, where *n* is the number of observations and *k* is the number of variables, we want to find the $s\times k$ Principal Component matrix $\textit{C}$, with usually $s<<k$ such that the projected data matrix $\textit{W}=\textit{X} \textit{C}^T$, also called scores matrix, will have dimension $n\times s$, where s represents the number of principal components. The problem can be seen as:

$$\begin{aligned}&\mathop {\textrm{minimize}}\limits _{{\bf {C}}}\quad \quad {\Vert {\bf {X}}-{\bf {XCC}}^T\Vert _{F}^2}\\&\mathrm{subject \, to}\quad \quad {{\bf {C}}^T{\bf {C}}={\bf {I}}} \end{aligned}$$

where $\Vert \cdot \Vert _F$ is the Frobenius norm. We implement the model using the prcomp *R* package.

By construction, PCA produces a continuous output vector of size *n* for each of the *k* selected principal components, also known as scores vectors. This means that PCA is useful in reducing the initial space of considered variables, by building new components which exploit the correlation structure characterizing the data. Each component is linked to a specific and a priori unknown latent factor which measures a potentially interesting information. Thus, the components are mutually independent by construction and the identified latent factors, if meaningful from an interpretation point of view, can overcome the existing endogeneity issues. In the present study, we aim at extracting two principal components, where ideally each of them is linked to a specific strategy, either NPIs or vaccination roll-out. Indeed, European countries plus United Kingdom were totally free in choosing the best mix of countermeasures, so we will expect to see some countries resorting totally to NPIs, some to favor vaccination roll-out and some others to mix the two approaches. In this regard, we employ PCA to shed light on the countries’ behaviors by exploring the obtained principal components.

Since PCA does not naturally handle time series data, we applied the following strategy: for each month, from March 2021 to December 2021, we picked the value of the considered variables on the last day of the month. In this way, we were able to run one PCA analysis per month using the pandemic-related variables available for each country, e.g., administered vaccination doses and NPIs (mask usage, health monitoring, school closure, social distance, business restrictions). The choice of the reference day per each month has been assessed through a proper robustness analysis, to assure that our results do not greatly vary upon the chosen data point (day). Specifically, a window up to three days before and after the last day of the month has been set and evaluated. Moreover, since we are more concerned with the evolution over time of the different strategies put in place by countries, the choice of a different point in time, say mid of the month, would not substantially change the results apart from shifting back or forth the observation time.

Through this modeling strategy, we can monitor the evolution pattern month by month of both the principal components and the positioning of the countries. We recall that each principal component can be considered as a new variable obtained from the linear combination of the original ones. Thus, we can interpret the principal components based on such combinations.

To ease the comparison, we draw ten biplot representations, each referring to a specific month and country pair. The biplot is a common graphical display used to map at the same time the two first principal components (the two most important ones) and the relative scores associated with each and every data point, the countries in our case. A PCA biplot shows both PC scores of samples (dots) and loadings of variables (vectors-arrows). The further away these vectors are from a PC origin, the more influence they have on that PC. Loading plots also hint at how variables correlate with one another: a small angle between the vectors representing the variables implies a positive correlation, a large one suggests a negative correlation and a $90^\circ$ angle indicates no correlation between two characteristics. When the vectors diverge and form a large angle (close to $180^\circ$), the variables are negatively correlated concerning the considered principal component. By looking at the position of countries in the biplot, we can infer the propensity toward a specific principal component, similarities and dissimilarities among countries, and specific patterns of a single country or of a group of countries.

The results of PCA will then be interpreted looking at:

1. the variable composition—in terms of NPIs and vaccination roll-out—of the two components that explain the largest part of the variance will be revealed;
2. the variable weights (loadings), revealing how countries apply different NPI and vaccination strategies;
3. whether the impact of vaccination roll-out becomes dominant in one of the components. We started the observation period in March 2021 since vaccinations were hardly available before that time in most countries.

## Discussion

This study investigates whether country response strategies varied in contrast to each other once vaccinations were available and reveals which NPIs were applied. Overall, the linear combinations of the first two components explain between about 70% and 85% of the variance between countries strategies for every month apart from April, where the explained variance for both components is 62%. The fact that in each month at least three measures contribute with an amount of about 0.5 to the first principal component indicates that the country differences cannot be explained by one or two single measures, but nearly every measure was applied to a different extent across countries. On the contrary, the second component, explaining from 20% to 25 % of the variance across measures, is driven by SOC and vaccination roll-out from June to December 2021. Accordingly, country strategies can be most easily distinguished by these two measures and it can be seen that, especially from September until December, countries made different choices with respect to vaccination roll-out. However, the first component still consists of a mix of four to five measures, meaning that all NPIs were addressed, although some measures seemed to be less effective according to studies on the effectiveness of NPIs^4^.

At the beginning of 2021, vaccination roll-out was limited by the availability of vaccines, whose distribution was coordinated by the European Union, resulting in low differences in vaccination uptakes across countries apart from United Kingdom. Indeed, United Kingdom produced its own vaccines and, thus, was able to administer more vaccinations compared to other countries. In the second half of the year 2021, when the bottleneck of vaccines was over, vaccination strategies were rather different across countries, representing the fact that some countries relied on an early administration of a third dose due to waning immunity^11^, while others incremented the third dose administration with the Omicron variant advent at the end of the year 2021.

The negative correlation between SOC and HTM shown in Table 1 indicates that HTM, e.g., testing, was more important in lower social restrictions contexts. In fact, it is expected that gatherings represent a lower risk, if people are proven to be uninfected. On the opposite, the remaining all positive correlations show that the measures are used complementary rather than substituting. This result is in line with the recommendations of^6^ to apply measures together. Finally, the highest correlation found between SOC and BUS might be driven by the fact that restrictions to gatherings were applied in both the private and the business contexts. Moreover, such high correlation can be explained by the fact that governments and health authorities often implement social distancing and business restrictions simultaneously during outbreaks, to limit as much as possible person to person contact which represents the main virus spread mean.

Considering strategies from a country perspective, four groups of countries can be derived by their amount of applied measures. The first group, including Austria and Italy, heavily relied on policy measures in all months. The second group, composed by Germany, France and Portugal, relied on measures in some phases but relaxed them in spring and autumn. The third group, including Denmark, Spain and Czechia, only applied measures more rigorously than other countries in some months. Finally, the fourth group, represented by United Kingdom and Sweden, did not implement NPIs as strictly as other countries. However, United Kingdom distinguished itself with a rapid vaccination rollout during the considered period. Thus, while in 2020, when the SARS-Cov-2 pandemic started, countries applied quite similar policy measures, e.g., lock-downs, as shown in earlier studies^14,28^, one year later, when vaccinations were made available, national strategies were quite diversified.

To summarize, the performed PCA analysis enabled to disentangling NPIs and vaccination roll-out across countries to represent and analyze the different national strategies, going beyond mere descriptive statistics. This method allowed us to reveal which combinations of measures explained the largest part of the variance across countries. So far, the Oxford Government Tracker only revealed if a certain NPI was applied at a certain point in time on an ordinal scale, providing a poor representation of the differences across countries.

A limitation of the study is that PCA, not being a dynamic tool, might not capture situations in which measures were implemented for only a short period in the middle of the month. However, the mentioned robustness checks showed that this should have a limited impact on our results. In addition, it should be noticed that a reason for more severe measures in some countries might be a more vulnerable population in terms of the share of older adults or prevalence of diseases, as pointed out in^18^. The latter could not be addressed in this study, but might be the rationale for heterogeneous measures, at least in some cases. Future research should address the impact of population characteristics on the use of measures and, consequently, whether different measures are reasonable under specific circumstances.

Our results show that European countries chose different strategies in terms of NPIs one year after the outbreak of the SARS-Cov-2 pandemic, even though the effectiveness and socio-economic impact of policy measures were already evident a few months after the first waves. Our findings also show that the full set of NPIs was applied - even though with different intensity and timing across the countries and no one was completely abandoned, at least in the considered period. Similarly, vaccination strategies started to diverge once a sufficient amount of vaccinations were available and the question whether to administer a third dose arose.

Overall, based on our results, European countries did not coordinate their measures during the investigated period, until the end of 2021, what could have been effective to reduce the burden of the pandemic in Europe.

## Data Availability

The data used are available from the OurWorldInData data repository (https://github.com/owid/covid-19-data/tree/master/public/data).
